# Effects of Monomer Composition of Urethane Methacrylate Based Resins on the C=C Degree of Conversion, Residual Monomer Content and Mechanical Properties

**DOI:** 10.3390/polym13244415

**Published:** 2021-12-16

**Authors:** Codruta Sarosi, Marioara Moldovan, Andrada Soanca, Alexandra Roman, Timea Gherman, Ancuta Trifoi, Andrea Maria Chisnoiu, Stanca Cuc, Miuta Filip, Georgiana Florentina Gheorghe, Radu Marcel Chisnoiu

**Affiliations:** 1 Institute of Chemistry Raluca Ripan, Department of Polymer Composites, Babes-Bolyai University, 30 Fantanele Street, 400294 Cluj-Napoca, Romania; codruta.sarosi@gmail.com (C.S.); marioara.moldovan@ubbcluj.ro (M.M.); stanca.boboia@ubbcluj.ro (S.C.); miuta.filip@ubbcluj.ro (M.F.); 2 Department of Periodontology, “Iuliu Haţieganu” University of Medicine and Pharmacy, 15 V. Babes Street, 400012 Cluj-Napoca, Romania; andrada.popovici@umfcluj.ro (A.S.); alexandra.roman@umfcluj.ro (A.R.); 3 Research Institute for Auxiliary Organic Products—ICPAO S.A., 8 Carpati Street, 551022 Medias, Romania; pap_timea12@yahoo.com (T.G.); ancuta_trifoi@yahoo.com (A.T.); 4 Department of Prosthodontics, “Iuliu Hațieganu” University of Medicine and Pharmacy, 32 Clinicilor Street, 400006 Cluj-Napoca, Romania; 5 Faculty of Dental Medicine, Carol Davila University of Medicine and Pharmacy, 17-23 Calea Plevnei, 010232 Bucharest, Romania; 6 Department of Cariology, Endodontics and Oral Pathology, “Iuliu Hațieganu” University of Medicine and Pharmacy, 33 Motilor Street, 400001 Cluj-Napoca, Romania; marcel.chisnoiu@umfcluj.ro

**Keywords:** dental resin, dimethacrylates, PEG, residual monomer

## Abstract

(1) Background: This study investigated the influence of Bis-GMA, TEGDMA, UDMA, and two different polyethylene glycol (PEG)-containing, UDMO-based co-monomers on the Young’s modulus and flexural strength, degree of methacrylate C=C double bond conversion and residual monomer elution of experimental dental resins. (2) Methods: Urethane methacrylate-based monomer was synthesised via a radical chain growth polymerization mechanism using PEG in order to improve the mechanical properties. Dental resins were formulated using Bis-GMA, UDMA, or UDMO as base monomers combined with TEGDMA as a dilution monomer and DMAEM + CQ as the photo-initiator system. Degree of conversion (DC), mechanical properties, and residual monomer content of light-activated methacrylate resin formulations were evaluated and statistically analysed by ANOVA and a Tukey’s test. (3) Results: PEG-containing UDMO resins had lower Young’s modulus and elastic strength than UDMA-derived resin for all irradiation times. The highest DC (67,418%) was observed for the PEG-containing UDMO-based resin formulation when light cured for 40 s. For all samples, DC increased with the photo-polymerization time. The amount of residual monomer decreased after increasing the light-curing period from 20 to 40 s, resin with UDMO content 0.01 mol of PEG having the smallest amount of free eluted monomer. (4) Conclusions: A strong structure–property relationship exists in photo-cured dimethacrylate-based dental resins. The time and quantity of the photochemical initiation system can influence the physical–mechanical properties of the resins but also the monomers in their composition.

## 1. Introduction

The use of composite resins as restorative materials based on dimethacrylic monomers is one of the most important attempts in the field of dentistry [1]. The organic matrix is typically based on dimethacrylate resins [2,3], while fillers vary depending on size, shape, and morphology, each offering different properties depending on the properties that the dental resin must provide [4].

The most commonly used monomer in dental composites is bisphenol A-glycidyl methacrylate resin (Bis-GMA) [5]. Bis-GMA is characterized as a long, rigid molecule with reactive double carbon bonds on both ends, a highly viscous monomer due to its hydroxyl groups, which increase its polarity and cause strong intermolecular interactions. To overcome the disadvantages of low monomer mobility and reactivity resulting in decreased DC and crosslinking density, Bis-GMA has been partially replaced with the low viscosity and more flexible UDMA, and it was diluted with dimetacrilat de trietilen glicol TEGDMA [6,7,8].

Free radical chain polymerization of the organic phase, most commonly initiated by photoinitiators or a chemical initiator and co-initiator, leads to the formation of a crosslinked network of esters, urethanes, amide bonds, and van der Waals interactions [9]. The degree of conversion of the C=C double bonds is correlated with the most important characteristics of the materials, such as mechanical properties, volumetric shrinkage, wear resistance, water absorption, and monomer elution [10].

Many researches [11,12,13] focused on determination of the extent of polymerization by quantification of unreacted double bonds remained after photocuring process. A key factor affecting the photopolymerization efficiency of methacrylate resins is the initial resin viscosity, as it influences monomer mobility, hence their reactivity. Local viscosity of the resin system is directly affected by monomer composition and monomer structure.

According to previous studies [13,14], DC was increased from 30% to 60% when bisphenol A-glycidyl methacrylate (Bis-GMA) was replaced with the less viscous Triethylene glycol dimethacrylate (TEGDMA), due to the higher monomer mobility. TEGDMA, due to its higher mobility and lower molecular weight, elutes more quickly, exhibiting cytotoxic, genotoxic, mutagenic, and allergenic effects on dental pulp cells [15]. These results suggest, again, that increasing the diluent monomer content is not the best method to improve monomers and C=C double bond conversion.

More, Cebe et al. [16], studied the release of residual Bis-GMA, TEGDMA, Hydroxyethylmethacrylate (HEMA), and Bis-EMA monomers from six different commercial composite resin materials by elution in 75 wt.% ethanol/water solution. The results showed higher concentrations of the eluted TEGDMA than of the other monomers.

The same variation was found by Fonseca et al. [17] in a more recent research, following a study to determine the effect of the replacement of Bis-GMA with ethoxylated bisphenol-A dimethacrylate (Bis-EMA), Bis-EMA 30, urethane-dimethacrylat (UDMA), and UDMA-modified on the polymerization kinetics, degree of conversion, water sorption, and solubility of dental composites. They have reported the highest rate of polymerization for the UDMA/TEGDMA mixture, without significantly different water sorption values.

Vallittu et al. [18], studied the effect of polymerization temperature and time on the residual monomer content of acrylic resin and evidenced that, by changing the polymerization conditions, implementing different treating procedures subsequent to polymerization, such as immersing the resin in water at 37 °C for 24 h, reduced the residual monomer amount. Similar results were obtained by Charasseangpaisarn et al. [13] using shorter treatment times with the immersion of resin in water at 50 °C for 1 h, followed by ultrasonic treatment/ultrasonic cleaning.

The aim of the present study was to obtain and investigate dimethacrylic dental resins (Bis GMA, TEGDMA; UDMA) with different concentrations of polyethylene glycol (PEG) in the chemical composition of UMDO co-monomers. The elastic strength, Young’s modulus, conversion of the base monomer, and the residual monomer of these resins were determined. The influence of the light curing time for each concentration of urethane monomers was also investigated.

The null hypothesis of the current study is that the PEG concentration directly improves the properties of composite resins.

## 2. Materials and Methods

### 2.1. Materials and Instruments

The resin matrix used in this study was composed of urethane methacrylate monomer (UDMA), oligomers (UDMO1/UDMO2), 2,2-bis[4-(2-hydroxy-3-methacryloyloxypropyl)phenyl] propane (Bis-GMA), and triethyleneglycol dimethacrylate (TEGDMA) (Merck KgaA, Darmstadt, Germany). Urethane monomer and oligomers were synthesized by ICPAO S.A. (Medias, Romania) according to the procedure described below, and Bis-GMA was synthesized by ICCRR—Babeş-Bolyai University, Cluj-Napoca, Romania. Photo-copolimerization was carried out in the presence of camphoroquinone (CQ) combined with N,N′-dimethylaminoethyl-methacrylate (DMAEM) (Merck KgaA, Darmstadt, Germany) as photo-initiator system. Other diluting monomers were used such as polyethylene glycol (PEG, M_n_-300), isophorone diisocyanate, 2-hydroxyethyl methacrylate (HEMA, ≥97%), and dibutyltin dilaurate (≥97%) (Merck KgaA, Darmstadt, Germany). Hydroquinone (>99%) (Fluka Chemie GmbH, Buchs, Switzerland) was added to inhibit the radical polymerization of acrylic function.

### 2.2. Syntheses of Dimethacrylates

UDMA was synthesized in nitrogen atmosphere under continuous stirring from a mixture of isophorone diisocyanate (0.04 mol), HEMA (0.08 mol), hydroquinone (0.2 × 10^−3^ mol), and dibutyltin dilaurate as catalyst. HEMA was gradually added at 38 °C for 1 h to the mixture of isophorone diisocyanate, hydroquinone, and dibutyltin dilaurate, after which the mixture was continuously stirred at 45 °C for another 3–4 h.

Two dimethacrylate oligomers (UDMO-1 and UDMO-2) were prepared following the same procedure, by gradually adding 0.01 and 0.02 mol of PEG (M = 300), respectively, at 38 °C for 1 h to the mixture of isophorone diisocyanate (0.04 mol), hydroquinone, and dibutyltin dilaurate. After 1 h of stirring at 38 °C, 0.05 mol of HEMA (UDMO-1) and 0.04 mol of HEMA (UDMO-2) were added gradually, followed by 3 h of stirring at 40 °C.

The monomer and oligomers were collected as a transparent viscous material and stored in the dark before use.

### 2.3. Preparation of Experimental Resin Formulations

Urethane methacrylate monomer and PEG-containing oligomers were mixed together with Bis-GMA, TEGDMA, DMAEM, and CQ according to the composition presented in Table 1, in a dark room. All six systems were well-mixed at room temperature to obtain a homogeneous, colourless liquid mixture and were stored in the dark before use.

Dental resins were cured by the principle of photo polymerization, due to the photo-initiation system, which comprises a photo-initiator (CQ) and a tertiary amine (DMAEM). We used an LED-curing light Woodpecker (Guilin Woodpecker Medical Instrument CO. Guilin, China) with a constant intensity of 1000 mW/cm^2^.

### 2.4. In Vitro Analysis Methods

#### 2.4.1. Fourier Transmission Infra-Red Spectroscopy (FTIR)

The Fourier transform infrared (FT-IR) spectra of the synthesized urethane methacrylate and resin formulations were recorded using an FT-IR spectrophotometer (JASCO 610, JASCO International Co., LTD., Tokyo, Japan) with an attenuated reflectance accessory with one scan at a resolution of 4 cm^−1^. Scanning was carried out in the range 4000–400 cm^−1^ for each sample. The spectra were normalized considering an internal standard absorption band at 1695 cm^−1^.

#### 2.4.2. ^1^H-NMR Analysis

^1^H-NMR spectra of the new methacrylate monomer and oligomers were recorded at 20 °C using a Bruker Avance 300 spectrometer (Bruker, Ettlingen, Germany) operating at 600 MHz. Chemical shifts (δ) are presented in parts per million (ppm) and were determined using residual CDCl_3_ as internal standard.

#### 2.4.3. Measurement of Double Bond Conversion

The near IR technique has been used to measure the degree of conversion (DC) by comparing the height of the 1640 cm^−1^ peak corresponding to the HC=CH_2_ (vinyl) stretching vibration before and after light-curing of the obtained resin formulations. The absorption band at 1608 cm^−1^ corresponding to aromatic rings of Bis-GMA is used as a reference as it does not vary with photopolymerization [19,20]. Degree of conversion (DC) was calculated from the ratio peak height in monomeric (before light-curing) and polymeric states (after light-curing in a Teflon mould with 5 mm diameter and 1 mm thickness), according to the following equation [17,21]

(1) $$DC=\left(100-\left(\frac{heigh_{polymer}}{height_{monomer}}\times 100\right)\right)(\%)$$

where, *heigh_polymer_* = *heigh*_λ=1640_/*height*_λ=1608_ and *heigh_monomer_* = *heigh_λ_*_=1608_/*height_λ_*_=1640_.

For each resin formulation, five determinations of the double bonds before and after polymerization were performed, the results being subjected to the ANOVA and Tukey statistical analysis (significance level *α* = 0.05).

#### 2.4.4. Mechanical Properties

Each resin formulation was light-activated by LED-curing light Woodpecker in a Teflon mold in the form of a parallelepiped with a size of 2 mm × 2 mm × 25 mm. Twenty bar-specimens were prepared. The irradiation time for both sides was 20 s for ten specimens and 40 s for another ten specimens of each sample formulation. After polymerizationm the specimens were kept at 37 °C for 24 h.

A three-point bending test (span 20 mm) was carried out to evaluate the Young’s Modulus of bending (MPa) and elastic strength (MPa) according ISO 4049 [22] standard with a material testing machine (LR5k Plus, Lloyd Instruments, Bognor Regis, UK) with 5 kN cell load capacity at a crosshead speed of 0.5 mm/min.

The data was analyzed with ANOVA test and Tukey test for post-hoc comparison between sample groups; the significance level α = 0.05. The statistical analysis was performed with the Origin2019b Graphing&Analysis software (OriginLab, Northampton, MA, USA).

#### 2.4.5. Residual Monomer Determination

Each specimen disc (15 mm diameter and 1 mm thickness, polymerized in five points with LED curing light Woodpecker) (L.1.1–L.3.2) was broken into small pieces and weighted using a digital scale and placed into a volumetric flask provided with an electric heating system. Acetonitrile solution was used as extractive medium. Each flask was stirred for 8 h at 60 °C. The resultant slurry was separated from the discs by filtration. The extracts in acetonitrile were rota evaporated to dryness and resuspended at 2 mL of acetonitrile, filtered in 0.45 µm PTFE filters, and analysed by HPLC.

A stock solution of a mixture of standards (Bis-GMA, TEGDMA, and UEDMA) was prepared by dissolving the monomers in acetonitrile (HPLC grade, Merck) to a final concentration of 333 µg·mL^−1^. Four calibration curves solutions were prepared by dilutions of the stock solution in the range between 333 and 55 µg·mL^−1^. The linearity of the response to standards was established with four concentration levels, and the regression factors *R*^2^ were >0.998.

The residual monomer content of light-activated methacrylate resin formulations was carried out by HPLC Chromatograph (JASCO International Co., LTD., Tokyo, Japan) equipped with a HPLC pump, a ternary gradient unit, column thermostat, and UV/VIS detector. The samples were manually injected. For each specimen, three injections were performed, the resulting quantity being their means and standard deviations.

## 3. Results

### 3.1. Fourier Transmission Infra-Red Spectroscopy (FTIR)

**Figure 1:**
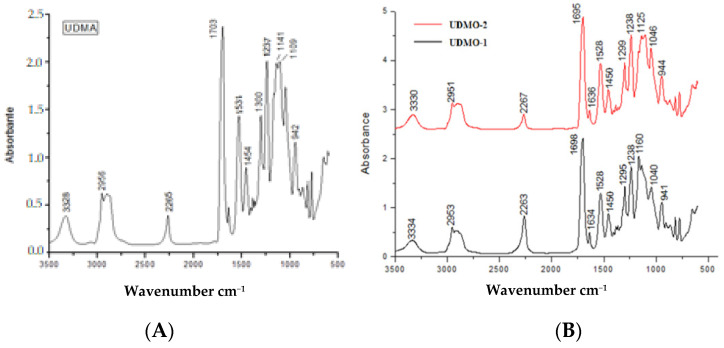
FTIR spectra of synthesized urethane dimethacrilate materials: (**A**) urethane dimethacrylate monomer and (**B**) urethane dimethacrylate oligomers.

Figure 1A shows the FT-IR spectra of the urethane dimethacrylate monomer (UDMA), and in Figure 1B the spectra of two oligomers with PEG in different ratios in the reaction mixture are presented. In Figure 1B, the spectra present significant differences in the 1690–2500 cm^−1^ domain regarding the intensities of the isocyanate characteristic band.

**Figure 2:**
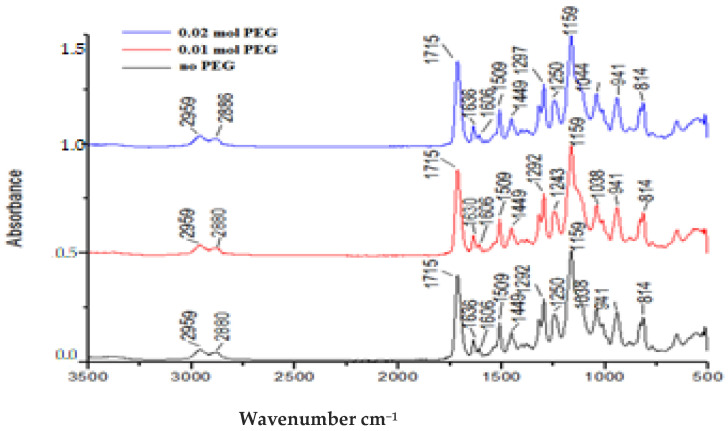
FTIR spectrua of urethane dimethacrylate resin: L.1.2 (0.02 mol PEG), L.2.2 (0.01 mol PEG), and L.3.2 (no PEG).

Figure 2 presents the spectra of prepared resin (Table 1) with different percentage of the PEG.

### 3.2. ^1^H-NMR Analysis

**Figure 3:**
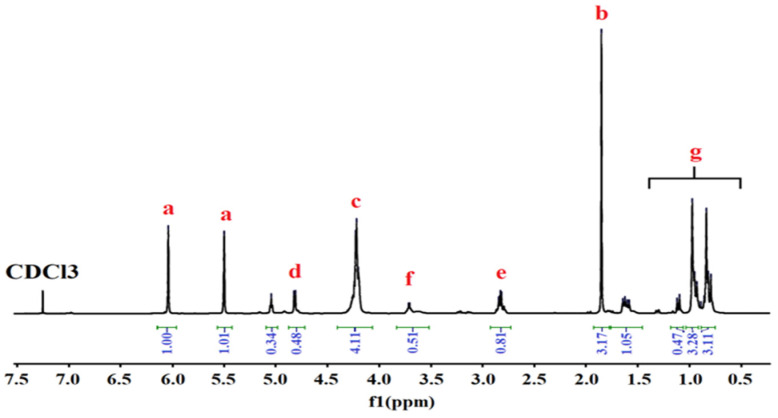
^1^H-NMR spectra of UDMA.

**Figure 4:**
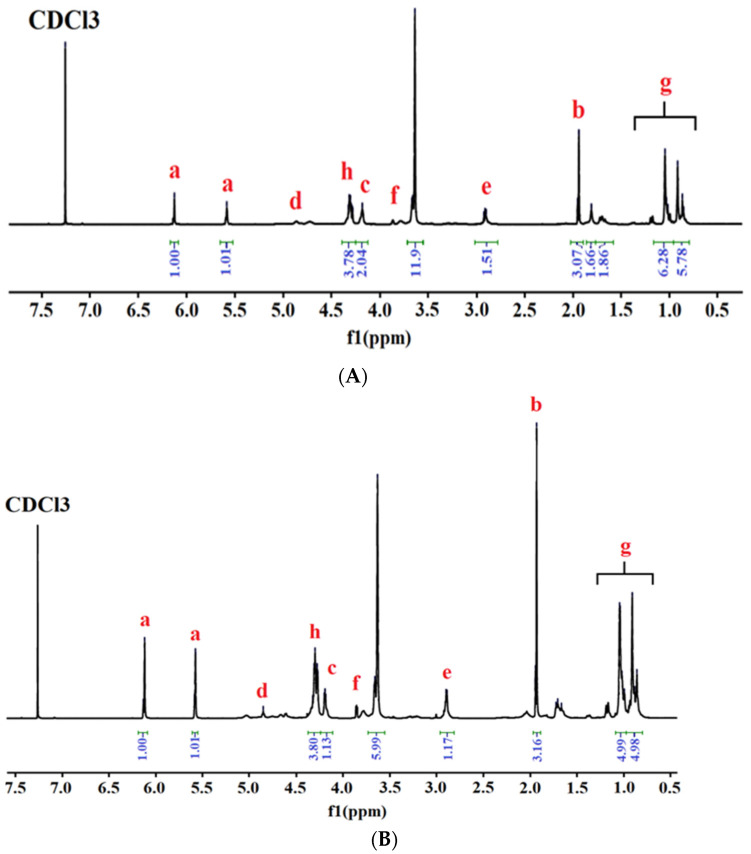
^1^H-NMR spectra of synthesized urethane methacrylate oligomers: (**A**) UDMO-1 and (**B**) UDMO-2.

^1^H-NMR analysis re-confirmed the structure of the synthesized urethane dimethacrylate monomer and oligomers, identified by FTIR analysis, according to the spectra presented in Figure 3 and Figure 4.

### 3.3. Double Bond Conversion

The increased value of DC in case of resin formulations obtained after a longer light-curing period is due to the higher rate of polymerization. As presented above, L.1, L.2, and L.3 resins have different monomer composition regarding the PEG content of the UDMA base monomer. As can be observed from Table 2, the introduction of PEG in the urethane dimethacrylate monomer composition led to higher DC values in the case of a longer curing time period.

Since the effectiveness of photopolymerization and percentage saturation of double bonds is quantitatively measured by DC, an increase in double bond conversion is expected in the case of a higher (40 s) curing time.

### 3.4. Mechanical Properties

**Figure 5:**
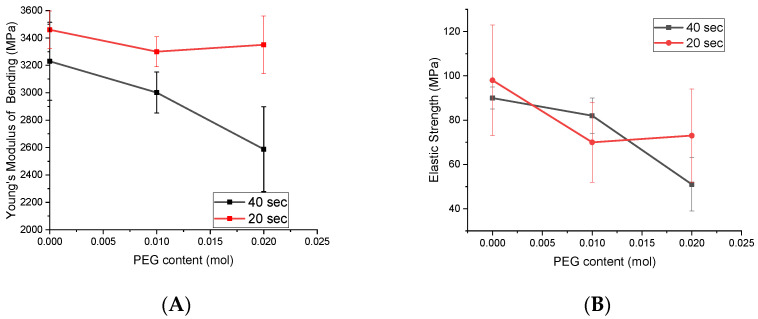
Effect of PEG content on the mechanical properties of Bis-GMA/TEGDMA/UDMA resins at different irradiation time on Young’s Modulus (**A**) and on elastic strength (**B**).

The effect of PEG content on the mechanical properties (Young’s Modulus (FM) and elastic strength (ES)) of Bis-GMA/TEGDMA/UDMA dental resins is presented in Figure 5.

### 3.5. Residual Monomer Determination

For Young’s Modulus, the ANOVA test obtained a value *p* = 0.09068, which indicates that there are no significant differences between the two groups of samples analyzed with different concentrations of PEG and different curing times. Regarding the Tukey’s test performed between all six groups with specimens, it was revealed that the group with the highest amount of PEG and 40s of polymerization is different from the rest of the investigated specimens.

To test the results of elastic strength, the ANOVA test showed that there are no significant differences between the two groups of samples with different polymerization times (*p* = 0.70667), but there are significant differences between samples with 0.02 mol of PEG and 20s polymerization time and the rest of the samples, after the Tukey’s test.

According to the data presented in the Table 3, the amount of eluted free monomer decreased after increasing the light-curing period from 20 to 40 s.

When PEG content of UDMO base monomer increase (L.3.), the extracted monomer amount is lower in case of 20 s curing time (L.3.1).

## 4. Discussion

The course of the copolymerization reaction between isophorone diisocyanate and HEMA was pursued through FTIR spectroscopy following the absorption of the isocyanate stretching band at 2265 cm^−1^, the reaction being considered complete after the disappearance of this band from the spectrum [23]. The urethane stretching band at 3328 cm^−1^ was also presented in the initial mixture composition. The C=O stretching band at 1695 cm^−1^ remains constant during polymerization and serves as an internal standard [24,25,26]. The decreased peak intensity from 2267 cm^−1^ attributed to the –N=C=O isocyanate group presented in the UDMO-2 (0.1190) and UDMO-1 (0.1586) spectra indicate a higher consumption degree of the isocyanate group through the polymerization reaction, suggesting that an isophorone diisocyanate:PEG = 4:1 molar ratio (UDMO-1) is not enough to complete the polymerization.

The null hypothesis was partially accepted, because resins with 0.01 mol PEG in composition (UDMO) had a lower Young’s modulus and elastic strength and smallest amount of free eluted monomer than UDMA-derived resin for all light curing time.

UDMO has a higher molecular methacrylate mass compared with UDMA but it is expected to exhibit superior reactivity due to the long chain of PEG that distances the polymer chains and eliminates steric impediments. PEG has been used as a plasticizer in the mixture of isophorone diisocyanate, HEMA, and TEGDMA by Buruiana et al. [27] in the process of methacrylate synthesis. PEG chemically reacts with the –N=C isocyanate double bond of the pre-polymer formed between 1 mol of HEMA and 1 mol of isophorone diisocyanate. UDMO has two isophorone diisocyanate molecules linked by the long chain of PEG. Therefore, isocyanate –N=C double bond conversion in the case of UDMO can be explained by the high reactivity of PEG due to the reactive marginal –OH functional groups.

In the process of urethane methacrylate (UDMA) synthesis, reagents conversion can be quantified by the isocyanate double bond consumption. After mixing the new UDMA or UDMO with Bis-GMA, HEMA, and TEGDMA and complete –N=C isocyanate consumption (as we demonstrated from the IR spectra of resin formulations—Figure 2), the quantification of methacrylate-derived vinyl HC=CH_2_ double bond conversion represented a great interest. The introduction of PEG in the urethane dimethacrylate monomer composition led to higher DC values in the case of a longer curing time period. This improvement in polymerization efficiency can be explained by the higher monomer mobility in case of UDMO-2 generated by the long chain of PEG that distances the polymer chains, increasing the mobility and reactivity of monomers. Even if UDMA has smaller molecular mass, compared with UDMO, it is a less reactive, more rigid molecule that increases monomer mixture viscosity and significantly affects polymerization efficiency.

According to other studies, the conversion of UDMA-containing polymer ranged from 76% to 87%, and it is significantly higher than the measures for Bis-GMA polymers.

A higher degree of conversion is associated with better mechanical properties and biocompatibility of the composites in terms of limited extractable free monomers. Any remaining unreacted or pendant C=C double bonds increase the final pool of leachable monomers [20].

All the ^1^H-RMN spectra confirmed the presence of signals corresponding to the resonance of the olefinic protons (a) (trans/cis: 6.10/5.60 ppm), methylidene protons of the urethane-ester (c) (4.2 ppm) groups, methylidyne protons near the urethane groups (f) (3.55 ppm), and those connecting to secondary nitrogen (e) (2.70 ppm). The signals of the methyl protons from HEMA and methyl protons from isophorone diisocyanate (b) and the hexadecyl chain (g) appeared at around 1.82−1.96 and 1.50−0.81 ppm, respectively. In all cases, formation of urethane is confirmed by the signal (d) at around 4.8 ppm, corresponding to the resonance of the proton linked to the secondary amine [23,26]. In the ^1^H-NMR spectra of urethane oligomers the presence of PEG is confirmed by the appearance of the signal (h) at around 4.4 ppm, corresponding to the resonance of the methylidene groups [23,26,28,29].

Mechanical properties of prepared resin were evaluated as function of chemical composition and curing time efficiency. PEG-containing UDMO-derived resins had lower Young’s Modulus (FM) and elastic strength (ES) than UDMA-derived resin for all light curing time, according to ES (ISO 40409), which should not be lower than 50 MPa. As a result of this, the flexibility of the polymeric network can be increased, leading to lower FM and ES. At the same time, when the PEG content is increased, we observed a slight increase of FM and ES at 20 s. The increase of FM is due to an increased crosslinking density by the new secondary interactions through an internal polymerization process by new hydrogen bonds. Thus, a resin material with improved strength but lower polymerization efficiency is obtained due to the unreacted monomers blocked in the secondary network structure.

It is suggestive that the more hydrophilic specimens, which have more superior relaxation ability, present lower flexural strength values, and this is a major determinant of the mechanical characteristics of resin materials [27]. The higher molecular weight co-monomer (PEG) has been reduced the resin shrinkage and stress generation due to their higher elasticity and the ability of the resin material to store elastic energy associated with recoverable elastic deformation [30,31,32]. Therefore, the polymerisation shrinkage and stress generation were reduced by using a higher molecular weight resin.

The amount of unreacted monomers trapped in the resin material varies with the type of monomers in the system and the degree of conversion after light cure.

The amount of eluted free monomer decreased after increasing the light-curing period. These phenomena can be explained probably by the increased photopolymerization efficiency and higher monomer conversion and degree of conversion of double bond at longer curing time.

When the PEG content of UDMO base monomer increases, the extracted monomer amount decreases. In correlation with the flexural properties presented above, this behaviour is due to the secondary network formation, which stiffens the structure and is trapped inside the unreacted monomers. Increasing the curing time, the photopolymerization degree is increased, the PEG molecules led to a more flexible polymer structure, and that increases the elution efficiency of unreacted monomers.

Since only the monomers that are not part of the polymeric network can be extracted from the polymeric mass, while the remaining groups are bound to the polymeric network, the ability to elute monomers from the material not only depends on the concentration of unreacted monomers but also on the structure of resins, the location of monomers within the polymer network, and the final network characteristics. In the case of L.3.2. resin, the increased amount of eluted monomers does not necessarily represent a higher amount of leachable monomers, but suggests the formation of a more elastic, without volumetric shrinkage stress resin formulation that does not block inside the unreacted monomers.

Monomer diffusion can be limited by the rigidity of the core structure imparted by the absence of PEG (plasticizing effect) in the case of L.1. and L.2. resin, whatever the curing time.

For all the tested monomers, the amount of extracted free monomers is in accordance with the maximum acceptable amounts of residual monomers remaining in resin according to ISO 20795-1 [33] and are set at 4.5 wt% for auto-curing and 2.2 wt% for light-curing resins. These values consider only the residual monomers in the resin and not their elution characteristics.

Ryta Łagocka [34] demonstrated that there are three main factors that influence the monomers’ release from polymerized dental resins: the degree of cure, the composition, and the size and chemical characteristics of the leachable components. Denis A.B. et al. [35] reported a higher amount of extracted TEGDMA residual monomer (0.493–1.031%) compared with Bis-GMA (0.267–0.915%) when extracted with acetonitrile, methanol, or ethanol (75%) solutions. Mehmet Ata Cebe et al. [16] a presented a higher concentration of the eluted TEGDMA than Bis-GMA, HEMA, and Bis-EMA from six different commercial composite resin materials. The higher concentration of eluted TEGDMA can be explained by its lower molecular weight and higher mobility, so it is eluted more quickly [34].

In contradistinction to these results, we obtained higher amounts for Bis-GMA than for TEGDMA. This is a positive aspect, because TEGDMA is a dangerous substance exhibiting cytotoxic, genotoxic, mutagenic, and allergenic effects.

Resin-based dental composites are widely classified based on the handling and composition of fillers [36]. It has been reported in many studies that the incorporation of nanoparticles into the resin-based dental material has improved bending strength and Young’s modulus, and most of the studies have shown that the incorporation of inorganic nanofillers into resins has improved some of the mechanical properties compared to unfilled resins [36]. Thus, the formulations tested after filling must meet the requirements of standard ISO 4049 [22] regarding the minimum bending strength for restoration materials, which can be used in previous or subsequent restorations. In addition to the degree of double bond conversion, which can be partially controlled by obtaining systems with medium viscosity (L.2.1.; L.2.2.), the residual monomer is also influenced by the same viscosity. Thus, these dental resins can be used depending on the filling for both conventional flowable or bulk-fill composites as long as an appropriate filling system is chosen [36]

It should be noted that this work had some limitations. Investigations have also been studied for dental resins without the addition of filler, which improves certain physical and mechanical performance. Currently selected formulations (both unfilled systems and those filled with different nano-powders) are being evaluated for the mechanical test protocol such as volumetric shrinkage, polymerization stress, and water sorption and solubility.

The novelty of this study consists in obtaining and investigating the resin matrix with two dimethacrylate oligomers, which can increase double bond conversion and reduce residual monomers after the polymerization process, even if the mechanical properties decrease, but not below the limits imposed for dental resins.

## 5. Conclusions

Considering the aim of this study, the following conclusions can be reached:

(1) Isocyanate double bond consumption in the process of urethane methacrylate synthesis can be improved by adding PEG in the optimal molar ratio of isophorone diisocyanate:HEMA:PEG = 2:2:1.
(2) Monomer conversion following the photopolymerization process affects both the strength and mechanical properties of final dental composites and also results in pendant methacrylate groups and unreacted monomers trapped in the material.
(3) The concentration of leached out monomer depends not only on the concentration of unreacted monomers but also on the morphology of the tested material and the associated differences in its chemical composition: the structural differences of the used base monomer, curing time, and final network characteristics.

## Figures and Tables

**Table 1:** Chemical composition of prepared experimental resin.

Resin Formulation	Monomer Composition (%)					Photoinitiator System (%)		Light Curing Time (s)
	UDMA	UDMO1	UDMO2	Bis-GMA	TEGDMA	DMAEM	CQ	
L.1.1.	20	-	-	45	33.5	1	0.5	20
L.1.2.	20	-	-	45	33.5	1	0.5	40
L.2.1.	-	20	-	45	33.5	1	0.5	20
L.2.2.	-	20	-	45	33.5	1	0.5	40
L.3.1.	-	-	20	45	33.5	1	0.5	20
L.3.2.	-	-	20	45	33.5	1	0.5	40

**Table 2:** Degree of double bond conversion for the prepared UDMA and UDMO-containing resin formulation at different curing time periods.

Resin Formulation	DC (%) ± SD
L.1.1.	56.704 ± 0.845 ^a^
L.1.2.	63.199 ± 1.006 ^b^
L.2.1.	44.585 ± 1.055 ^c^
L.2.2.	56.182 ± 1.014 ^a^
L.3.1.	56.157 ± 0.729 ^a^
L.3.2.	67.418 ± 0.553 ^d^
*p*	1.11793 × 10^−11^

Tukey test: groups of specimens marked with the same letter “^a^” show no statistically significant differences between them; the groups marked with different letters “^b^”, “^c^”, “^d^” show statistically significant differences between them. (*p* < 0.05).

**Table 3:** Residual monomer amount extracted in acetonitrile and quantified through the calibration curves described above.

Resin Formulation	Bis-GMA (%)	TEGDMA (%)	UDMA (%)
L.1.1.	0.586	0.246	0.332
L.1.2.	0.504	0.204	0.288
L.2.1.	0.524	0.216	0.307
L.2.2.	0.462	0.226	0.265
L.3.1.	0.536	0.276	0.284
L.3.2.	0.589	0.275	0.341

## Data Availability

Not applicable.

## References

[1] Iftikhar S., Jahanzeb N., Saleem M., ur Rehman S., Matinlinna J.P., Khan A.S. (2021). The trends of dental biomaterials research and future directions: A mapping review. Saudi Dent. J..

[2] Pratap B., Gupta R.K., Bhardwaj B., Nag M. (2019). Resin based restorative dental materials: Characteristics and future perspectives. Jpn. Dent. Sci. Rev..

[3] Yadav R., Kumar M. (2019). Dental restorative composite materials: A review. J. Oral Biosci..

[4] Kim K.-H., Ong J.L., Okuno O. (2002). The effect of filler loading andmorphology on the mechanical properties of contemporary composites. J. Prosthet. Dent..

[5] Pfeifer C.S., Shelton Z.R., Braga R.R., Windmoller D., MacHado J.C., Stansbury J.W. (2011). Characterization of dimethacrylate polymeric networks: A study of the crosslinked structure formed by monomers used in dental composites. Eur. Polym. J..

[6] Combe E., Burke F.J., Bernard T., Douglas W. (1999). Dental Biomaterials.

[7] Huang Q., Lin Z., Liang X., Liu F., He J. (2014). Preparation and characterization of antibacterial dental resin with UDMQA-12. Adv. Polym. Technol..

[8] He J., Söderling E., Vallittu P.K., Lassila L.V. (2013). Preparation and Evaluation of Dental Resin with Antibacterial and Radio-Opaque Function. Int. J. Mol. Sci..

[9] Peutzfeldt A. (1997). Resin composites in dentistry: The monomer systems. Eur. J. Oral Sci..

[10] Ilie N., Hilton T.J., Heintze S.D., Hickel R., Watts D.C., Silikas N., Stansbury J.W., Cadenaro M., Ferracane J.L. (2017). Academy of Dental Materials guidance—Resin composites: Part I—Mechanical properties. Dent. Mater..

[11] Ferracane J.L. (2011). Resin Composite—State of the art. Dent. Mater..

[12] Podgorski M. (2012). Structure–property relationship in new photo-cured dimethacrylate-based dental resins. Dent. Mater..

[13] Charasseangpaisarn T., Wiwatwarrapan C., Leklerssiriwong N. (2016). Ultrasonic cleaning reduces the residual monomer in acrylic resins. J. Dent. Sci..

[14] Leprince J.G., Palin W.M., Hadis M.A., Devaux J., Leloup G. (2013). Progress in dimethacrylate-based dental composite technology and curing efficiency. Dent. Mater..

[15] Rodríguez-Lozano F.J., Serrano-Belmonte I., Pérez Calvo J.C., Coronado-Parra M.T., Bernabeu-Esclapez A., Moraleda J.M. (2013). Effects of two low-shrinkage composites on dental stem cells (viability, cell damaged or apoptosis and mesenchymal markers expression). J. Mater. Sci. Mater. Med..

[16] Cebe M.A., Cebe F., Cengiz M.F., Cetin A.R., Arpag O.F., Ozturk B. (2015). Elution of monomer from different bulk fill dental composite resins. Dent. Mater..

[17] Fonseca A.S.Q.S., Moreira A.D.L., de Albuquerque P.P.A.C., de Menezes L.R., Pfeifer C.S., Schneider L.F.J. (2017). Effect of monomer type on the C=C degree of conversion, water sorption and solubility, and color stability of model dental composites. Dent. Mater..

[18] Vallittu P.K., Ruyter I.E., Buykuilmaz S. (1998). Effect of polymerization temperature and time on the residual monomer content of denture base polymers. Eur. J. Oral Sci..

[19] Manojlovic D., Radisic M., Vasiljevic T., Zivkovic S., Lausevic M., Miletic V. (2011). Monomer elution from nanohybrid and ormocer-based composites cured with different light sources. Dent. Mater..

[20] Douglas P., Albadarin A.B., Sajjia M., Mangwandi C., Kuhs M., Collins M.N., Walker G.M. (2016). Effect of poly ethylene glycol on the mechanical and thermal properties of bioactive poly(å-caprolactone) melt extrudates for pharmaceutical applications. Int. J. Pharm..

[21] Herrera-González A.M., Caldera-Villalobos M., Pérez-Mondragón A.A., Cuevas-Suárez C.E., González-López J.A. (2019). Analysis of Double Bond Conversion of Photopolymerizable Monomers by FTIR-ATR Spectroscopy. J. Chem. Educ..

[22] International Organization for Standardization (2000). ISO 4049:2000 Denstistry—Polymer-Based Filling, Restorative and Luting Materials.

[23] Liang X., Huang Q., Liu F., He J., Lin Z. (2013). Synthesis of novel antibacterial monomers (UDMQA) and their potential application in dental resin. J. Appl. Polym. Sci..

[24] Andreani L., Silva L.L., Witt M.A., Meier M.M., Joussef A.C., Soldi V. (2013). Development of dental resinous systems composed of bisphenol a ethoxylated dimethacrylate and three novel methacrylate monomers: Synthesis and characterization. J. Appl. Polym. Sci..

[25] Stansbury J.W., Dickens H.S. (2007). Determination of double bond conversion in a dental resin by near infrared spectroscopy. Dent. Mater..

[26] Liu D., Liu F., He J., Lassila L.V., Vallittu P.K. (2013). Synthesis of a novel tertiary amine containing urethane dimethacrylate monomer (UDMTA) and its application in dental resin. J. Mater. Sci. Mater. Med..

[27] Buruiana T., Buruiana E.C., Melinte V., Colceriu A., Moldovan M. (2009). Urethane dimethacrylate oligomers for dental composite matrix: Synthesis and properties. Polym. Eng. Sci..

[28] Willems G., Lambrechts P., Braem M., Celis J.P., Vanherle G. (1992). A classification of dental composites according to their morphological and mechanical characteristics. Dent. Mater..

[29] Alshali R.Z., Salim N.A., Sung R., Satterthwaite J.D., Silikas N. (2015). Qualitative and quantitative characterization of monomers of uncured bulk-fill and conventional resin-composites using liquid chromatography/mass spectrometry. Dent. Mater..

[30] Moldovan M., Balazsi R., Soanca A., Roman A., Sarosi C., Prodan D., Vlassa M., Cojocaru I., Saceleanu V., Cristescu I. (2019). Evaluation of the Degree of Conversion, Residual Monomers and Mechanical Properties of Some Light-Cured Dental Resin Composites. Materials.

[31] Floyd C.J.E., Dickens S.H. (2006). Network structure of Bis-GMA- and UDMA- based resin systems. Dent. Mater..

[32] Kerby R.E., Knobloch L.A., Schricker S., Gregg B. (2009). Synthesis and evaluation of modified urethane dimethacrylate resins with reduced water sorption and solubility. Dent. Mater..

[33] International Organization for Standardization (2013). ISO 20795-1:2013 Specification Dentistry—Denture Base Polymers.

[34] Łagocka R., Jakubowska K., Chlubek D., Buczkowska-Radlińska J. (2015). Elution study of unreacted TEGDMA from bulk-fill composite (SDR™ Dentsply) using HPLC. Adv. Med. Sci..

[35] Denis A.B., Diagone C.A., Plepis A.M., Viana R.B. (2015). The effect of the polymerization initiator and light source on the elution of residual Bis-GMA and TEGDMA monomers: A study using liquid chromatography with UV detection. Spectrochim. Acta A Mol. Biomol. Spectrosc..

[36] Lempel E., Czibulya Z., Kovács B., Szalma J., Tóth Á., Kunsági-Máté S., Varga Z., Böddi K. (2016). Degree of Conversion and BisGMA, TEGDMA, UDMA Elution from Flowable Bulk Fill Composites. Int. J. Mol. Sci..

